# Retrieving and Validating Leaf and Canopy Chlorophyll Content at Moderate Resolution: A Multiscale Analysis with the Sentinel-3 OLCI Sensor

**DOI:** 10.3390/rs13081419

**Published:** 2021-04-07

**Authors:** Charlotte De Grave, Luca Pipia, Bastian Siegmann, Pablo Morcillo-Pallarés, Juan Pablo Rivera-Caicedo, José Moreno, Jochem Verrelst

**Affiliations:** 1 Image Processing Laboratory (IPL), Parc Científic, Universitat de Valencia, 46980 Paterna, Spain; 2 Institut Cartogrfic i Geològic de Catalunya (ICGC), Parc de Montjüic, 08038 Barcelona, Spain; 3 Forschungszentrum Jülich GmbH, Institute of Bio- and Geosciences, Plant Sciences (IBG-2), D-52425 Jülich, Germany; 4 Instituto ITACA, Universidad Politécnica de Valencia, Camino de Vera s/n, 46022 Valencia, Spain; 5 CONACyT-UAN, Secretaría de Investigación y Posgrado, Universidad Autónoma de Nayarit, Ciudad de la Cultura Amado Nervo, 63155 Tepic, Nayarit, Mexico

**Keywords:** leaf chlorophyll content, canopy chlorophyll content, leaf area index, pixel heterogeneity, moderate spatial resolution, Sentinel-3, OLCI, FLEX, HyPlant

## Abstract

ESA’s Eighth Earth Explorer mission “FLuorescence EXplorer” (FLEX) will be dedicated to the global monitoring of the chlorophyll fluorescence emitted by vegetation. In order to properly interpret the measured fluorescence signal, essential vegetation variables need to be retrieved concomitantly. FLEX will fly in tandem formation with Sentinel-3 (S3), which conveys the Ocean and Land Color Instrument (OLCI) that is designed to characterize the atmosphere and the terrestrial vegetation at a spatial resolution of 300 m. In support of FLEX’s preparatory activities, this paper presents a first validation exercise of OLCI vegetation products against in situ data coming from the 2018 FLEXSense campaign. During this campaign, leaf chlorophyll content (LCC) and leaf area index (LAI) measurements were collected over croplands, while HyPlant DUAL images of the area were acquired at a 3 m spatial resolution. A multiscale validation strategy was pursued. First, estimates of these two variables, together with the combined canopy chlorophyll content (CCC = LCC × LAI), were obtained at the HyPlant spatial resolution and were compared against the in situ measurements. Second, the fine-scale retrieval maps from HyPlant were coarsened to the S3 spatial scale as a reference to assess the quality of the OLCI vegetation products. As an intermediary step, vegetation products extracted from Sentinel-2 data were used to compare retrievals at the in-between spatial resolution of 20 m. For all spatial scales, CCC delivered the most accurate estimates with the smallest prediction error obtained at the 300 m resolution (R^2^ of 0.74 and RMSE = 26.8 μg cm^-2^). Results of a scaling analysis suggest that CCC performs well at the different tested spatial resolutions since it presents a linear behavior across scales. LCC, on the other hand, was poorly retrieved at the 300 m scale, showing overestimated values over heterogeneous pixels. The introduction of a new LCC model integrating mixed reflectance spectra in its training enabled to improve by 16% the retrieval accuracy for this variable (RMSE = 10 μg cm^–2^ for the new model versus RMSE = 11.9 μg cm^-2^ for the former model).

## 1 Introduction

The world’s continued demographic growth together with the climate crisis put increasing pressure on the global food supply [1,2]. In this context, determining the photosynthetic potential and the primary production of vegetation is crucial, as it underlies the whole consecutive food chain [3]. At the basis of the photosynthetic process, the chlorophyll (Chl) pigments, Chl a and Chl b, are responsible for the conversion of the light energy into stored chemical energy [4]. The leaf chlorophyll content (LCC) is therefore a key ecological variable that needs to be monitored consistently in space and time [5,6]. This variable can be remotely estimated using optical satellite sensors since it impacts the spectral signature of the observed surface in the visible part of the spectrum. Changes in LCC produce broad variations in the leaf reflectance and transmittance, which in turn influence the canopy reflectance. The latter is however also strongly affected by other factors, such as the canopy architecture, the Chl distribution in the canopy, the leaf area index (LAI), and the soil background, which mask and confound the changes caused by LCC [7]. These confounding effects can be partly accounted for by retrieving the total canopy chlorophyll content (CCC) rather than LCC. CCC is often approximated by the product of LAI and LCC [8,9], with LAI being defined as the total one-sided foliage area per unit of soil surface area [10]. Moreover, CCC is usually estimated with higher accuracy than LCC. For instance, in a heterogeneous Mediterranean grassland, CCC was estimated with higher accuracy than LCC and LAI using a look-up table approach to invert the PROSAIL model [11]. Similarly, the authors of [12] obtained superior performances in estimating CCC compared to LCC by means of different vegetation indices in potato fields located in the Netherlands. Also using vegetation indices, the authors of [13] managed to cut prediction errors by half when estimating CCC as opposed to LCC in sugar beet crops situated in France.

The remote monitoring of vegetation properties generally requires observations with a high spectral resolution and temporal frequency, which are provided by moderate spatial resolution sensors with a typical spatial resolution ranging from a few hundred meters to a few kilometers (e.g., 300 m for OLCI/Sentinel-3 or 1 km for MODIS/TERRA and PROBA-V) [14–16]. At such moderate resolution, the surface observed through the instantaneous field of view of the sensor may be very heterogeneous, i.e., composed of a mosaic of different object types, such as vegetation patches, bare agricultural fields and man-made structures [17]. All these different objects contribute to the pixel radiance, making the retrieval of vegetation properties from medium-resolution satellite observations complicated and therefore challenging.

A new moderate-resolution imaging spectroscopy satellite mission is upcoming by 2024: the FLuorescence EXplorer (FLEX) mission [18] of the European Space Agency (ESA). The mission is dedicated to the global monitoring of the chlorophyll fluorescence emitted by terrestrial vegetation at a spatial resolution of 300 m. FLEX will fly in tandem formation with Sentinel-3 (S3), the latter providing the ancillary data needed to characterize and interpret the fluorescence signal. In the framework of the tandem mission, retrieval models have been recently developed to estimate several essential vegetation variables, including LCC and LAI, from reflectance data measured by the Ocean and Land Color Instrument (OLCI) mounted on S3 [19]. These OLCI products are distinct from the official, already publicly available, Level-2 land products from OLCI [20], which are the OLCI Global Vegetation Index (OGVI) corresponding to the fraction of absorbed photosynthetically active radiation (FAPAR), and the OLCI Terrestrial Chlorophyll Index (OTCI), a proxy for CCC [21]. The newly developed OLCI retrieval models are based on a hybrid regression strategy whereby reflectance spectra simulated by the SCOPE (Soil Canopy Observation, Photochemistry and Energy fluxes) [22] radiative transfer model (RTM) are used to train a machine learning regression algorithm (MLRA) called Gaussian process regression (GPR) [23]. Compared to the conventional MLRAs such as neural networks, GPR offers several advantages, including a competitive computational performance, the provision of an uncertainty (or confidence) level for each per-pixel prediction, as well as some insights on the band relevance in the constructed model [24,25]. In [19], the GPR model performances were evaluated using simulated data in an end-to-end simulation framework [26] and obtained vegetation products from S3 reflectance data were successfully compared to corresponding Sentinel-2 (S2) vegetation products (coarsened to a 300 m spatial resolution) as derived from the L2B biophysical processor embedded in ESA’s Sentinel Application Platform Software (SNAP) [27]. The next step in the model evaluation should be to carry out an actual validation by comparison with reference data, which are usually measured in situ and are presumed to represent the “true” target value [28,29]. However, in situ point measurements cannot be easily compared to retrieved pixel values from a moderate-resolution satellite sensor. A method to spatially aggregate or up-scale (the scale being here understood as the geographic scale or spatial extent) the ground sampling points to the spatial scale of the satellite is required. A common approach consists in the calibration of empirical transfer functions that establish a relationship between local field measurements and radiometric data from high spatial resolution imagery (satellite or airborne). The resulting fine-scale reference maps are then upscaled to the moderate spatial resolution of the satellite product of interest to enable comparison [9,30–33]. Typically, the in situ measurements are performed within elementary sampling units (ESUs) that approximate the extent of one or more pixels of the high-resolution imagery, ranging most often from 10 to 30 m [34,35]. In the present study, the used validation data were gathered within ESA’s 2018 FLEXSense campaign, which collected very high spatial resolution airborne data (≤3 m) from the HyPlant DUAL sensor concurrent with ground-based point measurements on croplands around Jülich, western Germany. The campaign measurements were not executed over ESU’s but the very high spatial resolution of the HyPlant data enables a direct comparison between the ground measurements and the fine-scale estimations. This requires an adjustment of our OLCI GPR models so that they can be applied to the HyPlant image, which is characterized by both a high spectral and spatial resolution. The resulting fine-scale retrieval maps can then be validated with the in situ measurements, before being aggregated to the 300 m resolution of the S3 satellite for comparison with the OLCI products. A similar approach was used by [36] to validate the MODIS LAI product, whereby LAI ground measurements were first grouped in multi-pixel patches before their evaluation against fine-scale estimations from 30 m ETM+ data and their subsequent aggregation to the MODIS resolution. The aggregation step assumes that the retrieved variables have a linear behavior across different spatial scales.

In view of the above, the main objectives of the present paper are the following: (1) to validate our moderate resolution (300 m) LCC retrievals derived from OLCI reflectance data using in situ measurements and high-resolution aerial images (3 m). As an intermediary step, the SNAP vegetation products extracted from Sentinel-2 (S2) data were used to compare retrievals at the intermediate spatial resolution of 20 m; (2) to explore whether the up-scaling at canopy level by means of CCC or the inclusion of pixel heterogeneity in the retrieval process improve the moderate-resolution Chl retrievals; and (3) to analyze how our OLCI models behave across scales, by applying the models to both fine-scale (3 m) and moderate-scale (300 m) synthetic OLCI images, which were generated using the HyPlant DUAL data.

The remainder of the paper is structured as follows. In Section 2, an overview of the pursued workflow is given as well as a detailed description of the used remote sensing data and of the retrieval model characteristics. Section 3 presents the validation results, starting at the 3 m resolution and followed by the S3 300 m and the S2 20 m resolutions, together with the scaling analysis. We end this paper with a discussion (see Section 4) of the model retrieval performances at the different scales and of the limitations of the used validation strategy.

## 2 Methodology

**Figure 1:**
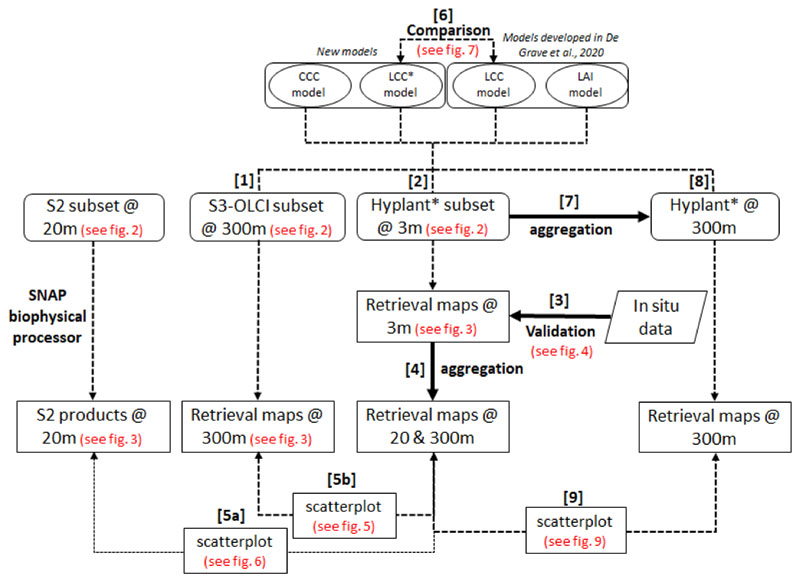
Schematic workflow of the presented methodology for the study of the retrieval of chlorophyll (Chl) content at moderate resolution. The development of the leaf chlorophyll content (LCC) and leaf area index (LAI) retrieval models is described in [19]. Two models were newly developed in the present study: the canopy chlorophyll content (CCC) model, by multiplying LCC and LAI before the Gaussian process regression (GPR) training, and the LCC* model, by integrating pixel heterogeneity. Hyplant*: Hyplant DUAL data resampled at the Sentinel-3 (S3)-Ocean and Land Color Instrument (OLCI) spectral resolution.

The pursued strategy for studying the retrieval of Chl content at moderate resolution is shown schematically in Figure 1. First, LCC and LAI models, as presented in [19], and a newly developed CCC model, were applied to a 300 m resolution S3-OLCI subset covering the area where field measurements were taken (see step [1] in Figure 1). To validate the obtained 300 m pixel values, the models were adapted in order to be applied to 3 m resolution HyPlant DUAL data (step [2]) and resampled at the S3-OLCI spectral resolution, and the resulting retrieval maps were then validated with in situ measurements (step [3]). The high-resolution estimations were subsequently aggregated to 20 and 300 m resolutions (step [4]) and compared with the corresponding S2 vegetation products (step [5a]) and the OLCI products (step [5b]).

Furthermore, a new OLCI model for estimating LCC was developed (LCC*) in view of accounting more effectively for the surface heterogeneity present at 300 m, and its performance was compared with the one presented in [19] (step [6]). The same hybrid approach as in [19] was followed, i.e., training of the GPR algorithm with simulated spectra generated by the RTM SCOPE. However, the new model also integrates reflectance spectra corresponding to mixed pixels (i.e., containing vegetated and non-vegetated areas) in the training database, and are therefore expected to improve the retrievals over heterogeneous pixels (see Section 2.3 below). Finally, with the intention to gain insight into how the retrieval models behave across different spatial scales, the high-resolution aerial image was aggregated to the S3 spatial resolution (step [7]) and the models were then applied to the aggregated image (step [8]). The derived retrieval maps were finally evaluated against the aggregated retrieved values (step [9]).

### 2.1 Remote Sensing Data

**Figure 2:**
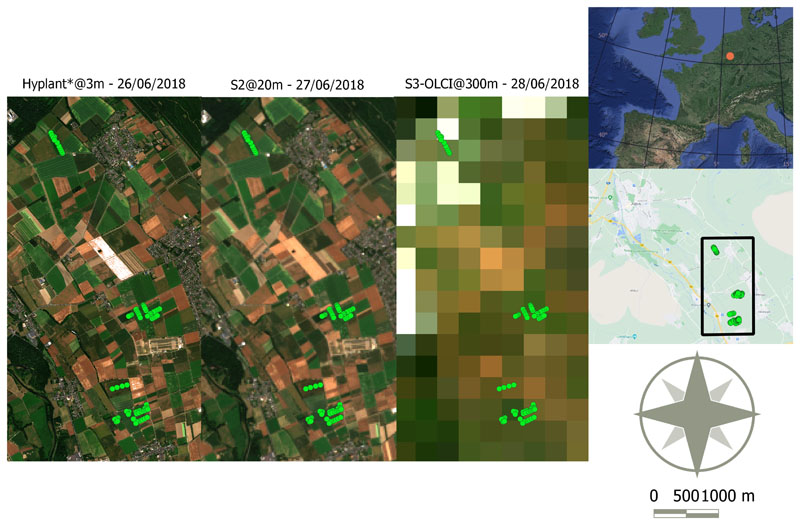
True-color composite (RGB: 665; 560; 490 nm) of the three used remote sensing datasets. (**Left**) the HyPlant* subset with a 3 m spatial resolution, which was resampled to the S3-OLCI spectral resolution (referred to as Hyplant*); (**middle**) the S2 subset with a 20 m spatial resolution; (**right**) the S3-OLCI subset with a 300 m spatial resolution. White pixels represent cloud pixels that were removed within the official S3 processing chain. The red dot on the map at European scale and the green dots on the maps of the study area indicate the localization of the in situ measurements.

General information on the used air- and space-borne imagery is summarized in Table 1. HyPlant DUAL is a hyperspectral airborne sensor consisting of two push broom imaging line scanners, which provide contiguous spectral information from 380 to 2500 nm, at a spectral sampling interval of about 1.7 nm in the visible and near-infrared and 5.6 nm in the shortwave infrared spectral domains. On the 26th of June 2018, HyPlant DUAL data of an area of approximately 15 × 10 km were acquired around the city of Jülich in the western part of Germany. The area is part of the Rur catchment and mainly consists of intensive agricultural fields and a large lignite open pit mine. In total, 18 flight lines, each of them having a spatial resolution of 3 m, were recorded and processed to top-of-canopy reflectance image products by applying the steps of radiometric, atmospheric, and geometric correction. Finally, the recorded flight lines were mosaicked to cover the entire area of interest. A detailed description of the HyPlant DUAL processing scheme can be found in [37]. Synthetic S3-OLCI reflectances were obtained at a 3 m spatial resolution as a linear combination of the Hyplant spectrum samples. Using Gaussian-shaped hyperspectral filters as basis functions, the weighted coefficients were extracted from the best fit of the S3-OLCI Spectral Response Function (SRF) by minimizing the sum of the squared residuals. For clarity, the resampled Hyplant data are labeled with an asterisk in the remainder of this paper. In order to perform a valid comparison between the HyPlant* and the S2/S3 imagery, cloud-free (or poorly cloud-covered) captures of each satellite sensor were selected, as close as possible to the HyPlant DUAL acquisition date. Regarding S3, the SYNERGY L2 reflectance product [38] was used, which combines the acquisitions of both optical instruments of S3, OLCI and the Sea and Land Surface Temperature Radiometer (SLSTR). Since the bands related to SLSTR were discarded in the present study, the SYNERGY product is hereafter referred to as the “OLCI reflectance product”. In fact, only 16 out of the 21 OLCI bands (see Table 1) were used since the five remaining bands are specifically designed for the characterization of the atmosphere (retrieval of cloud top pressure, water vapor, and aerosols) [39]. An OLCI reflectance product from the 28th of June 2018 over Western Europe was used in the present study. As for S2, Multi-Spectral Instrument (MSI) reflectance data of the 27th of June 2018 were downloaded from the Copernicus Open Access Hub (https://scihub.copernicus.eu/, accessed on 28 April 2020) and converted into vegetation biophysical products using the S2 Toolbox integrated in the SNAP software. The toolbox includes the L2B biophysical processor, which enables to map different canopy biophysical variables, including LAI and CCC, by means of hybrid models trained by neural networks [40]. From these two products, an unofficial S2 LCC product was calculated by dividing CCC by LAI. For all datasets, a subset covering the field measurements was used in order to limit the number of studied pixels and to enhance the processing speed. Figure 2 shows the subsets at the three studied spatial resolutions (3, 20, and 300 m) and enables to appreciate how the surface objects cannot be differentiated anymore at the 300 m resolution of S3. Regarding the S3-OLCI subset, a few pixels were contaminated by clouds and were therefore removed (thus appearing as white pixels).

### 2.2 In Situ Measurements

In parallel to the airborne data acquisition, LCC and LAI of different crops, namely maize, sugar beet, potato, and winter wheat, were measured non-destructively at several locations of the study area (see Table 2). In the different crop fields, samples were collected on transects covering the intra-field variability, with a distance of 10 to 15 m between each sampling point. All measurements were conducted between the 26th and the 29th of June 2018. At each location, a SPAD-502 Plus Chlorophyll meter (Konika Minolta Inc., Tokyo, Japan) was used to carry out 10 measurements from the upper leaves of different plants within an area of one square meter, which were subsequently averaged. Additionally, the LAI was measured within the same square meter using a SunScan photometer (Delta-T Devices, Cambridge, UK), with three below- and five above-canopy readings being collected at each sampling location. The CCC measurements, on the other hand, were computed by multiplying the LCC and LAI indirect measurements as was done in earlier studies (e.g., [41]). Since for winter wheat, LCC was not measured and only four LAI measurements were realized, this crop was discarded from the present study. Common Garmin hand-held GPS devices were used to measure the geolocations of the sampling points, which have quite low absolute accuracies (±5 m). Some of the GPS measurements were pointing at the tractor paths between crop rows, and the concerned point measurements were therefore manually moved to the middle of the crop rows. The ground measurements were used to validate the fine-scale estimations obtained from the HyPlant* dataset. Instead of comparing the measurements with single pixel values, the average of the pixel estimations falling within a 9 m diameter buffer, centered at each field sample, was taken. This enables to account for potential geolocation errors in both data sources.

### 2.3 Retrieval Models

The used vegetation retrieval models are based on a hybrid regression approach, which combines the generalization capabilities of physically-based RTMs with the flexibility and computational efficiency of machine learning [42]. The hybrid method has been elaborated in [19], and the key steps for developing the models are here briefly summarized. First, the RTM SCOPE was used to simulate 3000 surface reflectance spectra corresponding to a large variety of canopy realizations, at the spectral resolution of the OLCI sensor (16 bands). The used ranges for the input variables of the SCOPE model can be found in Appendix A, Table A1. Wavelength-dependent and -independent Gaussian white noise was added to the simulated reflectance spectra in order to make them more realistic and to integrate uncertainty sources [43,44]. Because SCOPE is only able to simulate vegetation spectra, 300 spectra corresponding to several types of non-vegetated surfaces (i.e., bare soil, artificial surfaces, etc.) were collected from S3-OLCI images and added to the SCOPE simulations. The resulting database was then used to train the GPR algorithm. As detailed in [19], the radial basis function (RBF) was used for the covariance matrix estimation, whose hyperparameters are obtained by maximizing the marginal likelihood of the observations. With the ambition to further improve the existing GPR models, two new GPR models were developed in the present study, i.e., CCC and LCC*. The model for estimating CCC was constructed by simply multiplying LCC and LAI before the GPR training. By contrast, the new LCC model (LCC*) was built by integrating synthetic spectra corresponding to mixed pixels in the training database [9,45]. Pure vegetated spectra, with no contribution of the background soil, were simulated by SCOPE using the same input variable ranges as for the other models (see table in Appendix A (Table A1)) except for LCC (15–100 μg cm^–2^) and LAI (3–10 m^2^ m^–2^). Pure non-vegetated spectra were collected from the HyPlant* DUAL image at 3 m resolution over bare fields and man-made surfaces. In order to create the mixed spectra, the reflectance at each wavelength, R(*λ*), was expressed as a linear combination:

(1) R(λ)=Rveg(λ)*v Cover + Rsoil (λ)*(1−v Cover ),

where *Rveg(λ)* refers to the reflectance of the pure vegetated spectra at wavelength *λ*, *Rsoil(λ)* is the reflectance of the pure non-vegetated spectra, *vCover* is the fraction of pure vegetation, and 1 – *vCover* is the fraction of non-vegetated surfaces in the observed pixel. The term *vCover* was randomly selected between 0 and 1 for each pixel. As for the other models, 300 bare soil spectra from S3 were added to the training database. The models were adapted before being applied to the resampled HyPlant* DUAL image. The S3 bare soil spectra of the training database were replaced by bare soil spectra collected directly from the HyPlant* image itself. The model performances were evaluated based on the usual goodness-of-fit statistics, being the coefficient of determination (R^2^), the root mean squared error (RMSE) and the normalized RMSE (NRMSE, in %, where the RMSE is normalized by the range of observed values).

### 2.4 Scaling Analysis

As a final exercise, the Hyplant* imagery was upscaled to the S3 spatial resolution and the impact of the upscaling on the vegetation properties estimations was assessed. The main goal of this analysis is to find out to which extent the land-cover heterogeneity within each pixel is going to bias the variable retrievals while avoiding discrepancies linked to the use of different datasets. Hence, the same models were applied to the HyPlant* DUAL data at two different spatial resolutions: at the original 3 m resolution and at the 300 m resolution after image rescaling. The image was upscaled to the S3 nominal spatial resolution by aggregating the finer pixels onto the sampling grid defined by the S3 acquisition detailed in Table 1. For this purpose, the Align Rasters tool, which is available in the QGIS software, was used [46].

## 3 Results

**Figure 3:**
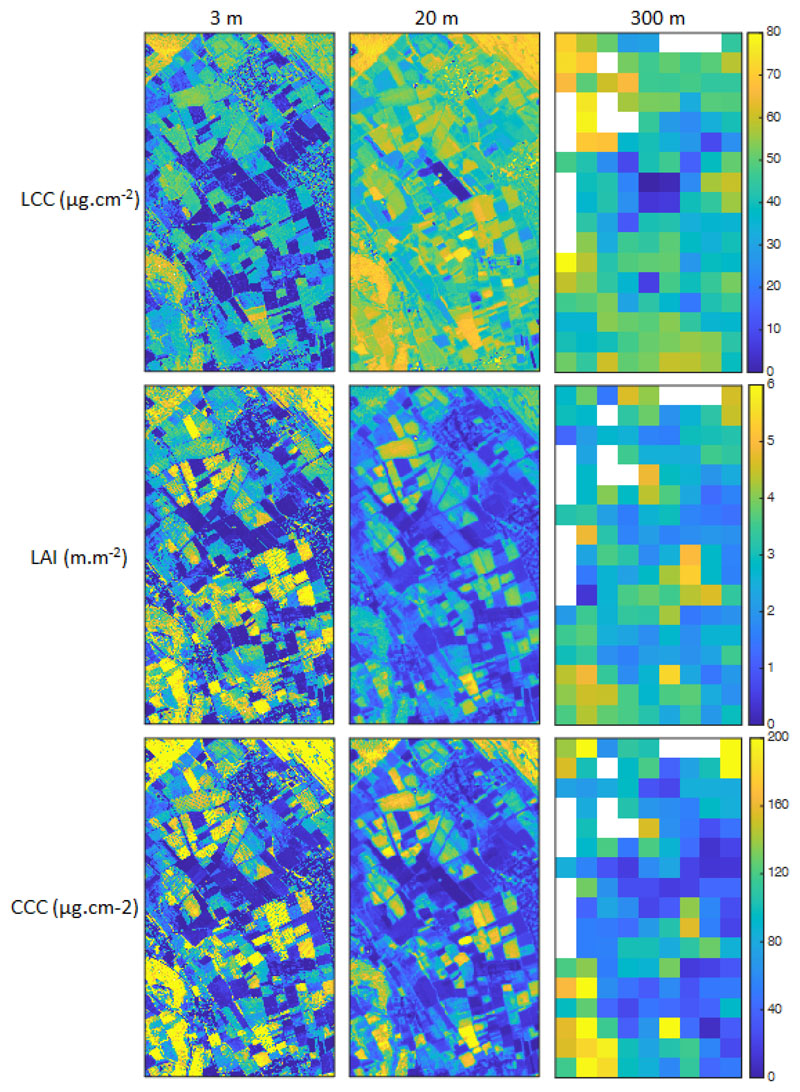
Variable maps (LCC, LAI, CCC) at the three spatial resolutions (see Figure 1 for location). First column: Retrieval maps obtained by applying our adapted OLCI models to the Hyplant* subset; second column: S2 SNAP vegetation products; third column: Retrieval maps obtained by applying our models to the S3-OLCI subset. White pixels represent either cloud pixels that were removed within the official S3 processing chain or pixels that were encountered as cloud-contaminated and removed within this study.

Our OLCI vegetation products at 300 m resolution, the corresponding S2 SNAP products at 20 m resolution, and the maps resulting from the application of our adapted GPR models to the HyPlant* reflectance data are displayed in Figure 3. Overall, the values are within consistent ranges and in line with earlier reported maps at other locations [19]. Similar patterns appeared, although the degradation of the spatial information due to the coarsening is evident at 300 m. When comparing the maps at 3 m versus the S2 products at 20 m, LCC and LAI exhibit relatively important differences while the two CCC maps show better agreement. The S2-LCC product displays higher values than the HyPlant-LCC product, whereas for LAI, a reverse pattern can be observed, with lower values at the coarser resolution as opposed to the finer resolution. The substantial higher values of the S2-LCC product are complicated to comment upon since we derived it ourselves from the S2 official LAI and CCC products. This only suggests that combining arithmetically retrieval maps is not a correct way to proceed.

### 3.1 Model Validation

**Figure 4:**
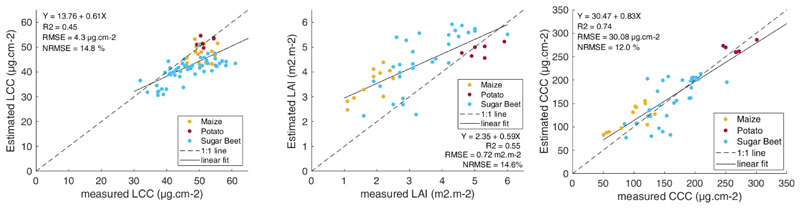
Validation of the fine-scale variable estimations at 3 m spatial resolution. Estimated values were generated by applying the retrieval models to a subset of a 3 m resolution HyPlant DUAL image. In situ measurements were gathered over crop fields (sugar beet, maize, and potato) near Jülich (Germany) and compared to the average of the pixel estimations falling within a 9 m diameter buffer, centered at each field sample.

**Figure 5:**
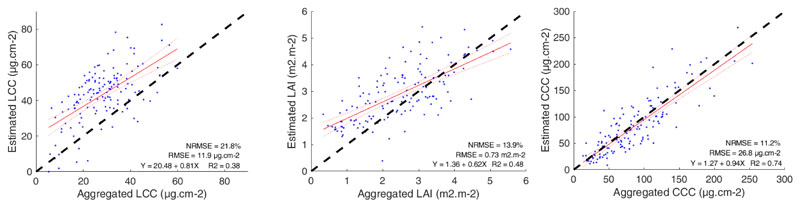
Validation of the OLCI vegetation products at a moderate scale. Estimated values were generated by applying the OLCI retrieval models to a S3-OLCI subset. Measured values were obtained by aggregating the fine-scale estimations retrieved from Hyplant* to the 300 m spatial resolution of S3-OLCI.

Direct validation was performed on the 3 m resolution of the HyPlant DUAL image to facilitate a best possible match with the field measurements. Good agreements can be observed between estimated and measured values for the three variables (see Figure 4). While the prediction errors were relatively low for all variables (RMSE = 4.3 μg cm^–2^ for LCC; RMSE = 0.7 m^2^ m^–2^ for LAI; RMSE = 30.1 μg cm^–2^ for CCC), the highest consistency was found for CCC with a correlation of R^2^ = 0.74 (versus R^2^ = 0.45 for LCC and R^2^ = 0.55 for LAI). Given the acceptable validation results, the Hyplant vegetation maps were subsequently used as a reference for validating the 300 m OLCI vegetation products. The fine-scale estimations were therefore aggregated to a 300 m spatial resolution and evaluated against the retrieved values derived from the OLCI reflectance product (see Figure 5). Two alternative aggregation methods were explored, i.e., using a simple averaged versus a LAI-weighted aggregation of LCC (results not shown). The former led to a higher correlation with the LCC values retrieved from the S3 imagery and was therefore selected. At the coarsened scale of 300 m, LCC suffered a remarkable drop in estimation accuracy with a more than twofold increased error (11.9 μg cm^–2^ versus 4.3 μg cm^–2^). Furthermore, a systematic overestimation across the whole considered range can be observed. This mismatch suggests that our OLCI LCC model requires further development, i.e., a retraining of the GPR algorithm with an adapted training dataset. Regarding the other variables, however, the estimation error was comparable for LAI (0.73 m^2^ m^–2^ versus 0.72 m^2^ m^–2^) and even improved for CCC (26.8 μg cm^–2^ versus 30.1 μg cm^–2^), as opposed to the finer scale. Again, the highest correlation appeared for the CCC variable with a R^2^ = 0.74 (versus R^2^ = 0.38 for LCC and R^2^ = 0.48 for LAI).

**Figure 6:**
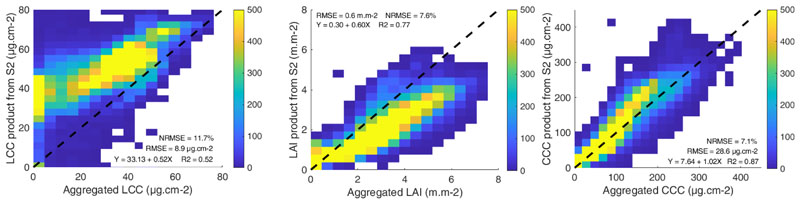
Comparison of the aggregated fine-scale estimations from Hyplant* with the corresponding 20 m resolution S2 products. Only LAI and CCC are products provided by the S2 ToolBox, whereas LCC was calculated by dividing CCC by LAI.

In order to explore an intermediary spatial resolution, the aggregated fine-scale retrievals at 20 m were compared to the S2 vegetation products as provided by the SNAP biophysical processor (see Figure 6). A similar trend can be observed for LCC and for the coarser scale of 300 m, with a systematic overestimation of the estimated values compared to the aggregated 3 m estimations. Although the prediction error was quite low for LAI, the aggregated values derived from the high-resolution HyPlant* data were systematically higher than the S2-LAI product. Again, the most consistent results were found for the CCC variable. Although here, a compensation effect probably occurs between the underestimation of LAI and the overestimation of LCC, resulting in improved estimations for CCC.

### 3.2 LCC Model Comparison

**Figure 7:**
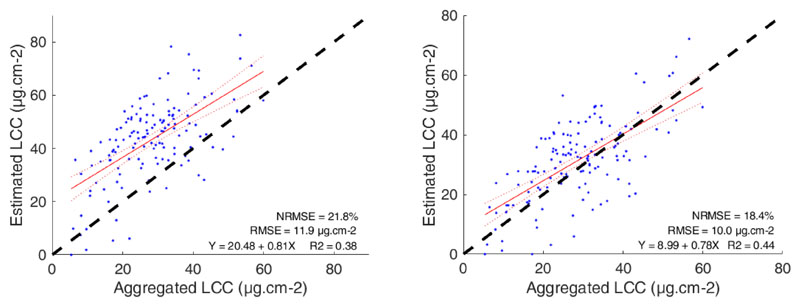
Comparison of the performances of two retrieval models estimating LCC. On the left, the OLCI model developed in [19] (LCC) and, on the right, the newly developed OLCI model integrating pixel heterogeneity (LCC*).

In an attempt to enhance LCC retrievals over moderate-resolution pixels, which often encompass heterogeneous areas, a new LCC* GPR model was trained and evaluated. Its performance compared to that of the original model has been considerably improved, although a still relatively high estimation error was observed (10 μg cm^–2^ versus 11.9 μg cm^–2^ for the old model; see Figure 7). The LCC* model also behaved somewhat more consistently, with a correlation of R^2^ = 0.44 (versus R^2^ = 0.38 for LCC model). More importantly, the LCC* model did not show a systematic overestimation over its whole range.

### 3.3 Analysis of Prediction Uncertainties

**Figure 8:**
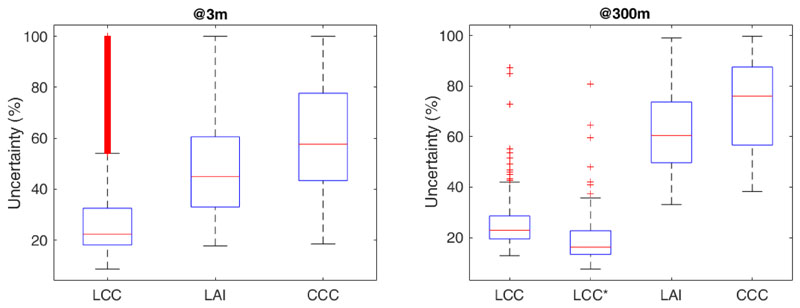
Comparison of the relative uncertainties, as defined by the coefficient of variances (%), at the spatial resolutions of 3 m (**left**) and 300 m (**right**). At the coarser resolution, the LCC model developed in [19] (LCC) and the newly developed model integrating pixel heterogeneity (LCC*) are compared. Data points beyond the whiskers are displayed with a red “+”.

An important advantage of the GPR method is that it provides uncertainties associated with the pixel estimations, either absolute uncertainties approximated by the standard deviation around the predictive mean, or relative uncertainties computed by the coefficient of variance (expressed in %). Figure 8 investigates the uncertainties associated with our retrieval maps (see Figure 3) and whether they remain constant across scales. Since the S2 vegetation products are derived by a neural network, they do not include such confidence intervals, and were therefore not integrated in the analysis. It can be noted that the uncertainties remain consistent across scales for LCC, the variable also exhibiting the lowest values. The newly developed LCC* model enables to further reduce the uncertainties (19.8% compared to 26.3% for the LCC model). It strengthens our confidence in the validity of this model for further usage. For LAI and CCC, however, uncertainties increased when moving from the high to the lower spatial resolutions.

### 3.4 Scaling Analysis

**Figure 9:**
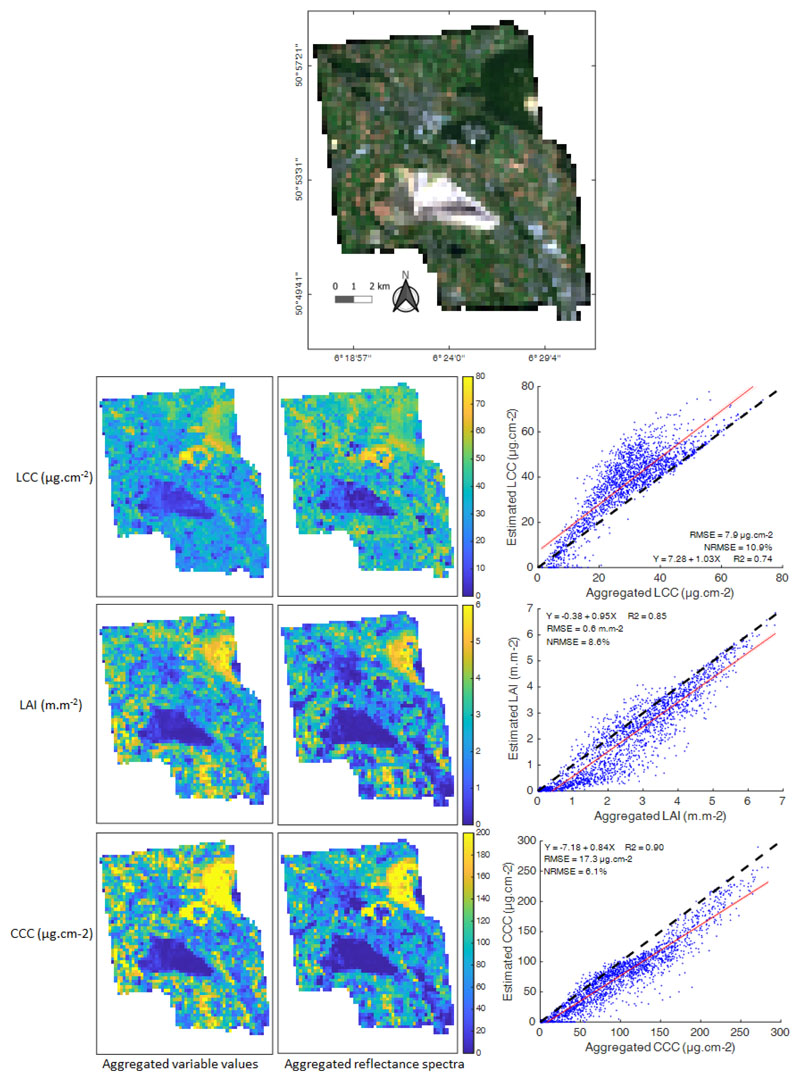
Comparison between estimated values derived from the application of the retrieval models to Hyplant* observations at a moderate scale (300 m) and at a fine scale (3 m). First column: Fine-scale estimations from the 3 m Hyplant* data were aggregated to 300 m; middle column: Moderate-scale estimations from the 300 m aggregated Hyplant* data; right column: Scatterplots between retrieval maps from the two first columns. The RGB (665; 560; 490 nm) image is the entire Hyplant image rescaled at a 300 m spatial resolution.

Finally, a scaling analysis was conducted in order to assess the impact of 3-to-300 m image coarsening. For this purpose, the vegetation parameters were retrieved from synthetic S3-OLCI reflectance data (derived from the HyPlant data) at 3 and 300 m using the same OLCI models. Under the assumption that the studied vegetation variables behave linearly across scales, the value of a variable estimated by a scale-invariant model at 3 m but aggregated to 300 m should approximate the estimation provided by the same model when applied to the 300 m averaged reflectance spectra. Figure 9 shows, however, that for all three variables, only the homogeneous areas were maintained in both retrieval maps, i.e., the forested areas located in the northeast of the image, characterized by the highest variable values, and the bare soil areas of the large pit mine, with null values. The intermediate values, which correspond mainly to heterogeneous pixels, were either overestimated, for LCC, or underestimated, for LAI and to a lesser extent CCC, when derived from aggregated reflectance spectra compared to the aggregated fine-scale estimations. Some of the underlying scaling mechanisms are briefly discussed in the following section.

## 4 Discussion

In the framework of the future FLEX mission, OLCI vegetation products were developed, and this work presents the first validation exercise based on in situ data coming from the 2018 FLEXSense campaign. Here we focus on LCC retrieval, since it is a variable that is complicated to retrieve from satellite data. To our knowledge, no operational product estimating LCC from space has been released so far. While LCC has been rather easily retrieved from leaf-level reflectance measurements since decades [4,47,48], at the satellite level, however, multiple factors influence the observed signal and the used retrieval method has to be able to disentangle them. At the moderate resolution of S3 and the future FLEX satellite (i.e., 300 m), another challenge arises regarding the validation of the retrieved pixel values and how to spatially scale ground-based point measurements to the scale of the satellite. A multiscale validation approach consisting of two consecutive steps was followed in the present study in order to validate S3-OLCI vegetation products: (1) very high-resolution estimations from airborne data were validated against in situ measurements, and then (2) upscaled to the spatial resolution of S3 as a reference to assess the quality of the moderate-resolution products.

Some limitations and inherent assumptions associated with the used validation method are worth discussing. First, it has to be kept in mind that the difference in data processing between the Hyplant DUAL data and the S3-OLCI data has evidently some influence on our results. Furthermore, although the obtained relative estimation accuracies at 3 m resolution were below 15% for the three variables, one could argue that the agreement with the in situ measurements is not good enough for LCC (R^2^ = 0.45) and LAI (R^2^ = 0.55) to be used as a reference for evaluating the coarser satellite products. While in situ measurements are considered as the true target values, their acquisition is also prone to errors and they are therefore intrinsically associated with uncertainties. The magnitude of these uncertainties depends on the applied measurement protocol inclyding the sampling scheme, i.e., determining the appropriate number and spatial extent of the field measurements. The first step towards a representative number of samples is the creation of a suitably sized ESU [28]. Unfortunately, the well-established ESU scheme was not followed during the 2018 FLEXSense campaign and consequently, a direct comparison between our very high resolutions estimations and the ground sampling points was performed. Evidently, this can lead to less reliable validation results in case the accuracy of the spatial registration or of the GPS readings was insufficient. Additionally, the multiscale validation approach assumes that the used model is scale-independent since the same models (although somewhat adapted in terms of added bare soil spectra) were applied to images from two different spatial resolutions. Different radiative and spatial processes play a role at the distinct spatial scales and conclusions based on one scale may not be applicable to another scale [49,50]. For instance, the role of vertical variability, or of canopy structure and associated shadowing effects, is more prominent at 3 m than at 300 m [49,51]. Related to that, several field instruments, among them the SunScan photometer used in this study, estimate LAI indirectly by measuring light transmission and using Beer–Lambert’s law or gap fraction theory [52]. These instruments generally underestimate the LAI values in clumped canopies (e.g., tree crowns in forests or crop rows in agricultural fields) since they assume a random spatial distribution of the canopy foliage instead of taking into account the dependent spatial dispositions of twigs and leaves. As a result, an effective quantity rather than the true LAI is measured [53]. The same is true for our LAI model based on SCOPE simulations, given that this RTM integrates a simple description of canopy architecture, approximated by a turbid medium, and assumes homogeneity in the horizontal direction. Our validation results at 3 m resolution (see Figure 4) show that our LAI model globally delivers higher values than the the SunScan photometer measurements. Since both are indirect methods used over clumped canopies, we can reasonably ask ourselves whether it is the SunScan that underestimates the true LAI values or our OLCI model that overestimates them. The comparison against the S2 LAI product however seems to confirm that our LAI model is subject to some overestimation, although more analysis is needed to evaluate the extent of the overestimation and whether the whole LAI range is concerned.

The presumed scale-independence of our retrieval models implies that the retrieved variables exhibit a linear behavior across different spatial scales, i.e., that the biophysical variable value at the coarse resolution amounts to the arithmetic mean of the biophysical variable at the finer resolution. The 3-to-300 m scaling exercise demonstrates, however, that this is in fact only true for pure pixels, either purely vegetated or purely bared, this being mostly visible within the large homogeneous areas of the region of interest, e.g., the northeastern forests and the pit mine (see RGB image in Figure 9). The heterogeneous pixels of the area, which have by definition intermediate variable values, were either overestimated (LCC) or underestimated (LAI and to a lesser extent, CCC), reducing the global retrieval performance. This matter was actually already investigated 20 years ago by Chen [54], who concluded that mixed land cover types in a pixel are the major problem causing scaling errors when deriving surface parameters, whereas the scaling effect can be ignored for pure pixels. Moreover, the authors of [17] stated that to limit the influence of spatial heterogeneity on non-linear estimation processes of land surface variables from remote sensing data, the proper pixel size must be small enough to capture the spatial variability of the vegetation cover at landscape scale while minimizing the spatial heterogeneity within the pixel, i.e., less than 100 m. This means that at larger pixel sizes, a correction based on the quantification of the intra-pixel heterogeneity should be applied, which can be characterized, e.g., by using land-use maps or multi-angular measurements [55]. Adjusting for scaling bias is mostly critical on crop sites, which are the most heterogeneous sites at the landscape level [56]. Since the SLSTR sensor of S3 measures oblique bands with a viewing zenith angle of 55° [39], their integration in our retrieval models could be a means to account for the scaling effect, at least partly. The added value of these bands will be tested when the reported inconsistencies in the SLSTR processing (e.g., co-registration issues between SLSTR and OLCI and calibration residual errors in the SLSTR SWIR bands [57]) are fixed.

According to our results, CCC is the variable presenting the most linear behavior across scales and this could explain why the retrieval of this variable was more successful than LCC (and LAI) at both spatial scales. However, the integration of synthetic mixed reflectance spectra in the model training enabled us to improve appreciably retrieval accuracies of LCC over heterogeneous pixels, which were systematically overestimated by the original LCC model. These results suggest that it could be an effective way to enhance LCC retrieval over heterogeneous pixels. While CCC was retrieved with higher accuracy than LCC, the estimations also came along with higher uncertainty levels associated with the retrieval maps (see Figure 8). This can be attributed to the fact that to obtain CCC, quantities with uncertainties were multiplied, which leads inevitably to the adding up of the relative uncertainties [58]. Yet, the GPR method can yield high relative uncertainties over low-valued pixels (i.e., bare soil or poorly vegetated pixels).

From a practical perspective, the provided uncertainties are useful to assess the retrieval performance in space and time [59]. If high uncertainties appear, ideally that implies that the training dataset should be augmented, e.g., by accounting for the surfaces where high uncertainty occurs. Hence, spatially-explicit information is provided to collect additional data to improve the robustness of the model. Another way to prevent obtaining high uncertainties over bare areas could be to use an alternative GPR algorithm, the variational heteroscedastic GPR (VHGPR) [60], that exploits the training with an additional step, leading eventually to lower uncertainties. In [61] for instance, VHGPR yielded consistently lower uncertainties than GPR at low LAI values. It was argued that since GPR assumes constant noise for all observations, it might not adjust well to the noise conditions at low variable levels. Thanks to its heteroscedastic character, VHGPR deals better with dependent signal-to-noise relations and is therefore certainly worth exploring in follow-up research. As a confirmation, the authors of [62] also preferred VHGPR above GPR for retrieving nitrogen content from corn and winter wheat crops. As such, improving the LCC model will involve adapting the training dataset as well as exploring whether alternative GPR methods may lead to lower uncertainties. Ultimately, once the models are considered sufficiently validated against in situ data, uncertainty estimates can function as a reliable source of information about the quality of the variable predictions and be used as a quality indicator, e.g., to mask out unreliable estimations.

## 5 Conclusions

In the framework of the FLEX/S3 tandem mission, hybrid retrieval models for estimating vegetation products from OLCI reflectance data were developed, and were subjected to a first validation exercise using in situ measurements collected during the 2018 FLEXSense campaign in croplands around Jülich in the western part of Germany. A multiscale validation strategy was pursued by using the in situ data to validate very high spatial resolution reference maps, which were subsequently aggregated to the S3-OLCI spatial scale in order to assess the quality of the OLCI vegetation products. Our results show that the retrievals of LCC and LAI are more sensitive to scaling effects than the retrieval of the combined variable CCC. Since these effects are caused by the presence of mixed surface types in moderate-resolution pixels, the retrieval of CCC tends to respond more robustly over heterogeneous areas. The adding of mixed reflectance spectra however enabled us to improve the retrieval performances for LCC. Further validation studies are needed to confirm these findings.

## Supplementary Material

Appendix A

## Figures and Tables

**Table 1:** Used imagery for the multiscale validation analysis. (*) For the purpose of the study, the Hyplant DUAL data were resampled to the S3-OLCI spectral resolution (referred as Hyplant*). MSI: Multi-Spectral Instrument. OLCI: Ocean and Land Color Instrument. S2: Sentinel-2.

Sensor	Acquisition Date	Spatial Resolution [m]	Bands	Spectral Range	Continuous Sampling
HyPlant* DUAL	26 June 2018	3	626	380–2500 nm	True
S2-MSI	27 June 2018	20	10	400–2200 nm	False
S3-OLCI	28 June 2018	300	16	400–1020 nm	False

Used S2-MSI bands: B2/490 nm; B3/560 nm; B4/665 nm; B5/705 nm; B6/740 nm; B7/783 nm; B8/842 nm; B8a/865 nm; B11/1610 nm; B12/2190 nm. Used S3-OLCI bands: Oa1/400 nm; Oa2/412.5 nm; Oa3/442.5 nm; Oa4/490 nm; Oa5/510 nm; Oa6/560 nm; Oa7/620 nm; Oa8/665 nm; Oa9/673.75 nm; Oa10/681.25 nm; Oa11/708.75 nm; Oa12/753.75 nm; Oa16/778.75 nm; Oa17/865 nm; Oa18/885 nm; Oa21/1020 nm.

**Table 2:** Descriptive statistics of the in situ measurements used for validation. LCC and LAI were measured in different crop fields situated near Jülich (Germany) by means of a SPAD-502 Plus Chlorophyll meter (Konika Minolta Inc., Tokyo, Japan) and a SunScan photometer (Delta-T Devices, Cambridge, UK), respectively. The CCC measurements were computed by multiplying the LCC and LAI measurements.

	LCC (μg cm ^–2^)				LAI (m^2^ m^–2^)				CCC (μg cm^–2^)			
	# Samples	Mean	Min	Max	# Samples	Mean	Min	Max	# Samples	Mean	Min	Max
Maize	15	50.7	46.2	55.6	11	1.8	1.1	2.5	11	95.1	50.8	134.5
Potato	6	51.8	49.2	54.4	6	5.2	4.6	5.9	6	267.1	246.0	300.9
Sugar Beet	51	46.6	31.9	61.0	30	3.5	1.6	6.0	30	161.2	85.1	252.0
Total	72	47.9	31.9	61.0	47	3.3	1.1	6.0	47	159.3	50.8	300.9
