# Supplementary material for: Retrieving and Validating Leaf and Canopy Chlorophyll Content at Moderate Resolution: A Multiscale Analysis with the Sentinel-3 OLCI Sensor
Source: Remote Sens (Basel). Author manuscript; Available in PMC 2022 Sep 7. (PMC7613399; doi:10.3390/rs13081419)
Supplement: Appendix A [file EMS152670-supplement-Appendix_A.pdf]

Appendix A

Table A1. The used ranges for the input variables of the SCOPE model. N: leaf mesophyll structure; Cab: leaf chlorophyll content; Cca: leaf carotenoid content; Cdm: leaf dry matter content; Cw: leaf water content; LAI: Leaf Area Index; LIDFa and LIDFb: parameters characterizing the leaf inclination distribution function (LIDF); SMC: Soil Moisture Content; BSM Bright, BSM lat and BSM lon are the three parameters characterizing the BSM (Brightness–Shape–Moisture) spectral soil model (for more information on the model, see [63]); SZA: Solar Zenith Angle; OZA: Observer Zenith Angle; RAA: Relative Azimuth Angle. * truncated Gaussian; ** constraint: Cw/(Cw + Cdm) between 0.45 and 0.93; *** constraint: $|\text{LIDFa}| + |\text{LIDFb}| \leq 1$.

Variable Type	Variable	Distribution	Min	Max	Mean	SD
**Leaf structure**	N	Gaussian *	1	2.7	1.5	0.5
	Cab (μg·cm^{-2})	Uniform	1	100		
	Cca (μg·cm^{-2})	Gaussian *	0	30	10	5
	Cdm (g·cm^{-2}) **	Gaussian *	0.002	0.02	0.005	0.003
	Cw (g·cm^{-2}) **	Gaussian *	0.005	0.035	0.012	0.006

Table A1. *Cont.*

Variable Type	Variable	Distribution	Min	Max	Mean	SD
**Canopy structure**	LAI (m^2 m^{-2})	Uniform	0.1	10		
	LIDFa ***	Uniform	−1	1		
	LIDFb ***	Uniform	−1	1		
**Soil**	SMC (%)	Gaussian *	5	55	25	12.5
	BSM Brightness	Gaussian *	0.01	0.9	0.5	0.25
	BSM lat (°)	Gaussian *	20	40	25	12.5
	BSM long (°)	Gaussian *	45	65	50	10
**Geometry**	SZA (°)	Uniform	0	80		
	OZA (°)	Uniform	0	25		
	RAA (°)	Uniform	0	180		
